# How to locate and appraise qualitative research in complementary and alternative medicine

**DOI:** 10.1186/1472-6882-13-125

**Published:** 2013-06-03

**Authors:** Brigitte Franzel, Martina Schwiegershausen, Peter Heusser, Bettina Berger

**Affiliations:** 1 Center for Integrative Medicine, Faculty of Health, University of Witten/Herdecke, Gerhard Kienle Weg 4, Herdecke D-58313, Germany

**Keywords:** CAM, Qualitative studies, Meta-Ethnography, Quality appraisal

## Abstract

**Background:**

The aim of this publication is to present a case study of how to locate and appraise qualitative studies for the conduct of a meta-ethnography in the field of complementary and alternative medicine (CAM). CAM is commonly associated with individualized medicine. However, one established scientific approach to the individual, qualitative research, thus far has been explicitly used very rarely. This article demonstrates a case example of how qualitative research in the field of CAM studies was identified and critically appraised.

**Methods:**

Several search terms and techniques were tested for the identification and appraisal of qualitative CAM research in the conduct of a meta-ethnography. Sixty-seven electronic databases were searched for the identification of qualitative CAM trials, including CAM databases, nursing, nutrition, psychological, social, medical databases, the Cochrane Library and DIMDI.

**Results:**

9578 citations were screened, 223 articles met the pre-specified inclusion criteria, 63 full text publications were reviewed, 38 articles were appraised qualitatively and 30 articles were included. The search began with PubMed, yielding 87% of the included publications of all databases with few additional relevant findings in the specific databases. CINHAL and DIMDI also revealed a high number of precise hits. Although CAMbase and CAM-QUEST® focus on CAM research only, almost no hits of qualitative trials were found there. Searching with broad text terms was the most effective search strategy in all databases.

**Conclusions:**

This publication presents a case study on how to locate and appraise qualitative studies in the field of CAM. The example shows that the literature search for qualitative studies in the field of CAM is most effective when the search is begun in PubMed followed by CINHAL or DIMDI using broad text terms. Exclusive CAM databases delivered no additional findings to locate qualitative CAM studies.

## Background

In medical and health service research during the last decade, the number of systematic reviews, meta-analyses and Health Technology Assessment (HTA) reports, specifically of randomized clinical trials (RCTs), has increased significantly. Currently, there are more than 4600 systematic reviews of quantitative studies alone available in the Cochrane Library [1]. In contrast, however, qualitative studies enjoy a greater popularity in the field of health service research. Qualitative studies are applied when methods are needed to specifically understand patients’ experiences and perceptions of healthcare and health care professionals [2-4]. They are applied when phenomena have to be explored, for gaining an understanding of social life, and for answering questions about the why and how of individual experiences [5-7]. Qualitative studies involve an interpretive approach [8], with greater openness and flexibility than quantitative studies [9-12].

They have become increasingly relevant to the development and evaluation of complex interventions [13]. Meanwhile, several methods have been established to synthesize qualitative research and to systematically include qualitative research results in medical decision making. Even though the methods of meta-synthesis are highly variable, qualitative meta-syntheses play a growing role in improving the understanding of patients’ perspectives [14].

Typically, medical and health service studies, including CAM research, use quantitative methods. While RCTs may be effective in determining the outcome of a treatment’s efficacy and safety on features of a disease, qualitative studies in CAM do not exert much influence in natural science research. However, the nature of clinical knowledge based on quantitative research methods is exposed to some limitations, for example, when phenomena or contexts of illness or health are to be investigated. Qualitative methods provide a more thorough approach for describing human behaviour [5]. In the context of CAM, qualitative studies allow a deeper understanding of subjectivity and complexity within human experience, turning them into a powerful tool for increasing our knowledge of important processes within CAM [5,15]. As the randomized controlled trial eliminates such additional or contextual factors methodologically, its feasibility for the evaluation of whole systems and individual aspects of human beings is limited. Hence, the methods of qualitative research can ideally be applied as a conceptual frame for “whole systems research” [16]. In summary, the value of the qualitative inquiry in CAM research lies in the in-depth understanding of complex individual details that are not captured by standardized methods [17,18]. However, there are specific challenges when conducting meta-syntheses including comprehensive literature searches in the CAM field as well as in the field of qualitative research. A quick search in PubMed is not considered adequate and may result in the introduction of bias into the findings of a review, and therefore more databases are needed for comprehensive searches [19]. The current PubMed CAM filter has several non-specific search terms that contribute to the lack of specificity of search output and that identify some irrelevant studies as “CAM”, such as plant-derived compounds in chemotherapy [20]. Specialized CAM databases may here provide the highest sensitivity and precision [19]. While searches in CAM are complex to begin with, the search for qualitative research adds still more challenges [21,22]. Qualitative research represents various research methodologies, including ethnography, phenomenology, grounded theory and narrative analysis, which might hinder retrieval depending on the database [23]. Qualitative systematic reviewers are therefore urged to search the literature systematically in an expansive manner and to enhance transparency of the complex literature search process by giving thorough explanations of their search strategies [21,24,25]. Over and above that, when it comes to the appraisal of qualitative studies, there is an on-going methodological debate about appropriate appraisal tools. A large range of different checklists are available in the literature [4,11,12,23,26-31]. The major differences are caused by the diversity of aims in educational, psychological, social and health disciplines. Furthermore, many reasons exist for the variety of different quality appraisals in qualitative research. For example, checklists have been adapted from quantitative criteria [12], have been an attempt to reply to the scepticism and distrust of qualitative work [32], and were created in competition for research funds [32]. In addition, as the world is often socially constructed and not compatible with objective standards, *sui generis* criteria or criteria idiosyncratic to specific studies were formulated, [10,11,33,34]. Finally, controversy has been caused by fundamental epistemological orientations [33] or checklists have been added with new criteria such as fairness, ethics, and saturation [34]. Numerous instruments exist and some checklists are yet to be validated or are not yet commonly used in practice.

The purpose of this publication is therefore to illustrate the process by which a literature search for a qualitative research synthesis in the CAM field was performed and to give an example of how a checklist was selected to appraise the retrieved studies. The underlying research topic was the concept of individualized medicine from the perspective of patients using CAM. There are only a few publications about qualitative research synthesis in CAM [35-38]. The CAM meta-ethnographies published so far have mainly focused on one treatment modality or on one indication. In this article, we outline a working example of how qualitative research in the field of various CAM treatments and indications can be conducted and which type of quality appraisal can be applied. Our case study in retrieving “qualitative studies” in “CAM” for a meta-synthesis has to our knowledge not previously been reported.

## Methods

The current study was performed in the context of our research topic “concepts of individualized medicine from the perspective of patients using CAM”. We performed a comprehensive literature search and appraisal for a meta-synthesis with qualitative studies. Qualitative studies reveal that the concept of individualized medicine is part of the common expectation of patients approaching CAM practitioners. Therefore, we expected detailed ideas about individualized medicine among patients using CAM. We searched for qualitative studies asking for patients’ reasons for seeking CAM therapies. We approached the research topic with a detour of searching for patients’ reasons for CAM use to find patients’ expectations of individualized medicine. The project included three sequences: 1. Systematic literature search and appraisal of selected publications of qualitative studies that investigated patients’ reasons for seeking CAM therapies; 2. Conduct of the meta-ethnography following Noblit and Hare’s [39] method to synthesize the key concepts of why patients are using complementary medicine; and 3. Interpretation of patients’ concepts of individualized care.

### Search methods

Sixty-seven electronic databases were systematically searched with a publication time frame between 1980 (we did not expect substantial qualitative research on CAM before 1980) and July 2011. The searches were performed in medical, social science, psychology, nutrition and complementary medicine databases to ensure that various CAM modalities and all aspects of patients’ reasons for seeking CAM were covered. We looked at the literature searching techniques of The Cochrane Qualitative Methods Group [40]. In our current literature search, several diverging techniques were applied in the beginning of the search to ensure that the resulting hits were highly comprehensive and that no studies were disregarded for the planned synthesis. Moreover, differing search terms were tested to achieve higher sensitivity for the retrieval of all potentially relevant studies. The databases that were included in the literature search, the search terms and search techniques are summarized in the first section of Table 1. The different techniques were not applicable for every database and had to be adjusted accordingly. During the search process, we tested whether specific key words that are usually used in qualitative research methodologies (e. g., “grounded theory”, “ethnography”), or broad-based text terms such as “qualitative research”, “qualitative study” and “interviews” either in thesaurus or free-text termini could be utilized (Table 1). Since the search “patients’ concepts of individualized medicine who are using CAM” revealed no findings, we approached the research topic with the detour of searching with different terms for patients’ reasons for seeking CAM as listed in Table 1. Additionally, we tested a string set of either multiple qualitative research search terms or “patients’ reasons” (OR “patients’ concepts” OR “patients’ expectations” OR “motivation” OR “attitude to health”) terms. The string sets were then interconnected with the broad terms “CAM” or “complementary and alternative medicine” AND “qualitative research” to identify relevant studies (Table 1). The literature search involved two members of the research group (BB, BF) with the help of a librarian/information specialist. The study selection was performed according to predetermined inclusion and exclusion criteria (Table 2). The determination and decision about the inclusion or exclusion of articles was initially conducted with the selected abstracts and thereafter using the full article. The full publications were read and a decision was made regarding which articles would be suitable for the qualitative appraisal in a joint session of same two members (BB, BF) of the research team. Reliability issues were addressed and resolved by discussion. The search and selection procedure was repeated in July 2011 again by one researcher (BF) to ensure that none of the relevant papers had been omitted.

**Table 1:** Meta-syntheses search strategy


**Databases**	API-on©, CAMbase, CAM-QUEST®, CINAHL, Cochrane Library, DIMDI, GREENPILOT, Heclinet, MedPilot, PubMed, Psyndex, PsynINFO, Sinbad, Somed, DIMDI included EMBASE, GLOBAL Health, Derwent Drug File, NHS Economic Evaluation Database, MEDLINE, ISTPB + ISTP/ISSHP, Derwent Drug File for PSYNDEX, SciSearch, Adis, Newsletters, Deutsches Ärzteblatt, SOMED, Social SciSearch, AnimAlt-ZEBET, ETHMED, Thieme-Verlagsdatenbank, PsycINFO, BIOSIS Previews, HECLINET, Thieme-Verlagsdatenbank PrePrint, AMED, CAB Abstracts, Health Technology Assess. Database, gms, Cochrane Centr. Reg. of Contr. Trials, CCMED, Hogrefe-Verlagsdatenbank u. Volltexte, gms Meetings, EMBASE Alert, Cochrane Datab. of Syst. Rev. Karger-Verlagsdatenbank, IPA, DAHTA-Datenbank, Krause & Pachernegg Verlagsdatenbank, Derwent Drug Backfile, MEDIKAT, Database of Abstracts of Reviews of Effects
	GREENPILOT included AGRIS, AGRICOLA, BMELV, BfR (Risikobewertung), CC GREEN, CC MED, DissOnline, Econis, ELFIS, Ernährung. Umwelt. Agrar., FLI (Tiergesundheit), IPB (Pflanzenbiochemie), IPK Gatersleben (Kulturpflanzen), JKI (Kulturpflanzen), MEDLINE, Medizin. Gesundheit., MRI (Ernährung und Lebensmittel), SSG Küsten- und Hochseefischerei, SSG Veterinärmedizin (TiHo), UBA-OPAC und ULIDAT, UFORDAT
**Search terms**	*Complementary and alternative medicine*
	(1a) Complementary and alternative medicine OR (1b) CAM OR (1c) complementary medicine OR (1d) alternative medicine
	*Qualitative research terms*
	(2a) qualitative research OR (2b) qualitative studies OR (2c) interviews OR (3) [exploratory OR grounded theory OR content analysis OR focus groups OR ethnography]
	*Patient decision making terms* (only in PubMed, CINHAL and GREENPILOT)
	(4) reasons OR (5) [concepts OR patient expectations OR motivation OR attitude to health OR patient communication OR health knowledge OR patient acceptance of health care OR patient participation OR physician-patient relations OR professional-patient relations OR socioeconomic factors]
**Search strategy**	(1) AND (2); (1) AND (2) OR (3); (1) AND (2) OR (3) AND (4); (1) AND (2) OR (3) AND (4) OR (5)

**Table 2:** Inclusion and Exclusion criteria

**Inclusion criteria:**	**Exclusion criteria:**
Qualitative studies with patients	Qualitative studies with therapists
Ethnographies	Perspectives of teaching personnel
Grounded Theory	Quantitative questionnaire studies
Content analysis	Mixed-Method studies with mainly quantitative studies
Peer reviewed Journals	Surveys
Published in English or German	Observational studies
All ethnic groups	Review and theory papers, study design, secondary analysis
Reasons for CAM	Randomized trials and outcome studies
Motivation to use CAM	HTA quantitative reviews
Patient perceptions of integrative medicine	Studies of patients’ information seeking of CAM
Patient perceptions of individualized medicine	Studies with conditions of CAM use
Patient / Therapist relation or interaction	Studies about questions of when and how CAM is used
Studies 1980-2011	Editorials
	Socioeconomic data analyses

### Quality assessment of selected qualitative studies

According to the Cochrane Qualitative Methods Group, the review teams should use a critical appraisal instrument that is underpinned by a multi-dimensional concept of quality in research and hence includes items to assess quality according to several domains, including quality of reporting, methodological rigour and conceptual depth and breadth [41]. In an attempt to find a standard checklist, the literature was searched in detail for the different checklists for qualitative research. Already published meta-synthesis examples were reviewed to see which critical appraisal check list had been used. Again, the variety here was large and almost every single meta-ethnography used a different non-validated or self-made checklist. Keeping this variety in mind and discovering that there was no standard checklist for performing meta-synthesis, the research team selected the checklists of CASP, Cohen & Crabtree, and the German checklists of Steinke and of Behrens and Langer in a narrow stage-by-stage selection [23,26-28].

Based on the widespread tradition of qualitative research in Germany, especially of qualitative research evidence based on the field of nursing, we decided to use the checklist of Behrens and Langer from the University of Halle [26]. As nursing science is a patient-oriented discipline, this checklist was most congruent with our research question and also added newer criteria such as saturation. The checklist was tested for feasibility by the qualitative research colloquium prior to the assessment. The nursing background, the 12 main questions and the assisting sub-questions were beneficial for our research question in CAM research. Table 3 lists the translated standardized questions from the checklist of Behrens and Langer and the number of (+)s indicates where the included studies fulfilled the criteria [42]. The checklist from Behrens and Langer was adjusted using other validated checklists from the literature [28,31,43]. Apart from the Behrens and Langer checklist questions, three core criteria that were repeatedly found in almost all German checklists and in the validated checklists were also added as comprehensive general questions: openness, inter-subjective validity and reflexivity [10-12]. These additional questions were added for an overall judgement for the appraisal of the included publications. The appraisal was performed by two researchers (MS, BB) involving a third co-reviewer (BB) in case of discrepancies. The appraisal group did not set criteria for the assignment of the grades. An inter-rater agreement for grades 1–6 was not possible. Thus we used nominal scales (+) and (−) to ensure a common scale and a clear assignment for the inter-rater agreement. For example, saturation was existent (+) or not (−), and the questions were open (+) or not (−). However, the grades were helpful to keep an overview of the overall quality of the 30 appraised articles (papers were rejected when the score was higher than 3.5). Reliability testing was performed through a communicative exchange at any one third of the appraised studies and we counted the concordance between both appraisals with (+) and (−).

**Table 3:** Translated questions according to checklist of Behrens and Langer with number of studies achieving criteria (+)

**Question:**		**Number of included studies with (+):**
1.	Is the research question clearly formulated?	27
2.	Which qualitative design was selected and was a reason given?	27
3.	Was a literature review performed?	16
4.	Were the participants adequately selected and the population clearly defined and was the selection justified?	29
5.	Were the participants and their environment and also the researchers sufficiently described?	14
6.	Was the data collection method described in detail?	24
7.	How was the data analysis performed?	28
8.	Was the data collection conducted until saturation had been reached?	17
9.	Are the results comprehensive, plausible and coherent?	28
10.	Were the results confirmed?	23
11.	Do the results help my own practice to better understand the participants in their settings?	30
12.	Are there concrete possibilities to transfer the findings to other settings?	28
Added by the general questions:		
A.	Openness	6
B.	Intersubjective validity	14
C	Reflexivity	8

## Results

### Database findings of qualitative CAM research

#### Search terms

This article demonstrates a case study of how qualitative research in the field of CAM studies was identified and critically appraised. The results of the main search strategies that were feasible for all selected databases are shown as a comparison in Table 4. Searching with “broad terms” was the most effective procedure. The search strategies with the terms “qualitative research”, “qualitative studies” and “interview” combined with “complementary and alternative medicine” were the only strategies that could be applied similarly for all databases. Specific MeSH (Medical Subject Heading) terms or methodological index terms that aid the identification of qualitative research (“exploratory”, “grounded theory”, “content analysis”, “focus groups” and “ethnography”) provided no additional results and delivered numerous false hits. Applying longer search strings with the intersection of different kinds of terms of “qualitative study” with “CAM” and with “patients’ reasons” terms turned out to be impossible in some databases. The databases revealed zero or numerous non-relevant hits, and therefore these could not be applied as a search strategy. In some databases like GREENPILOT or MedPilot, longer strings yielded a high number of hits with no relevance to the research question. In addition, the search term “qualitative research” revealed numerous hits with very low relevance. Overall, the search strategies as shown in Table 4 with broad search terms (“qualitative research” or “qualitative studies” or “interviews”) AND (“complementary and alternative medicine”) had the highest recall and precision. In most databases, the abbreviation term “CAM” and the search term “complementary therapies” resulted in a high number of hits with a very low precision and were therefore not suitable for the search. The specific CAM databases like CAMbase and CAM-QUEST® [44,45] delivered fewer hits than the medical or nursing databases. The search terms “CAM” or “complementary and alternative medicine” or “complementary medicine” or “alternative medicine” or “complementary therapies” pinpointed high specificity. In combination with “qualitative research”, the hits were sparse. An important finding in our search strategy was that the specialized “CAM” databases yielded only one relevant title each.

**Table 4:** Search results when applying broad terms

	**(Qualitative research)**	**CAm**	**(Complementary and alternative medicine)**	**(Qualitative research) AND CAM**	**(Qualitative research) AND (Complementary and Alternative Medicine)**	**Number of included publications in meta-ethnography (precision)**
PubMed	61.067	25.339	4.194	259	157	26
MedPilot	233.402	178.706	15.423	960	1.441	1
Cochrane library	662	86	321	14	1	0
CAMbase	349	183	280	1	0	0
CAM-QUEST®	1	15.856	43	1	0	0
API-on©	16	6	0	0	0	0
CINHAL	7.061	2.684	8.438	31	46	1
GreenPilot	265.822	255.664	8.230	34.147	1.422	0
Heclinet	7	0	0	0	0	0
Psydoc	10	33	0	0	0	0
PsynINFO	133	3	20	0	5	1
Sinbad	2	1	1	0	0	0
Somed	65	24	16	0	0	0
DIMDI incl. AMED	55.479	87.008	14.725	191	187	1

#### Suitable databases

The database that supplied the highest yield was GREENPILOT [46]. It is the virtual library for nutrition, environment and agriculture. GREENPILOT delivered multitudinous hits from the field of dentistry. Only a small number were relevant for the search project and many duplicates appeared in the matches, as sometimes the hits appeared up to four times. Although at first sight, GREENPILOT seemed to be a helpful database for the research question (with its scope for nutrition and environment including MEDLINE), it turned out to be less specific and helpful for the research question. Furthermore, MedPilot [47] delivered the second most hits; however, there was low specificity and many duplications. As a result of the findings mentioned above, the last three search strategies from Table 4 with the broad terms were analysed and duplicates were removed. The analyses for the relevant hits started with PubMed followed by Medpilot and the other databases as listed in Table 4. Additional relevant hits that were not available in PubMed were found only in MedPilot, CINHAL, PsychINFO and DIMDI. CINHAL and DIMDI would have been the other suitable databases for starting the search, because the hits were also very precise. In CINHAL, the abstracts were also easily obtainable. When comparing sensitivity after applying the search terms “qualitative research” AND “complementary and alternative medicine”, PsychINFO (20%) was the most effective, followed by PubMed (16%), CINHAL (2%), DIMDI (0.5%) and MedPilot (0.07%).

Overall, PubMed emerged to be the most suitable database for our research project, as the search with PubMed yielded 87% of the relevant included qualitative studies. The specificity was higher than in the other databases and abstracts could be screened easily with no admission rates. This made the search most efficient for our research question. The other databases that seem to be advisable for a research project on CAM and qualitative research are CINAHL, DIMDI (including PsycINFO®) and MedPilot (when only German publications are being searched).

**Figure 1:**
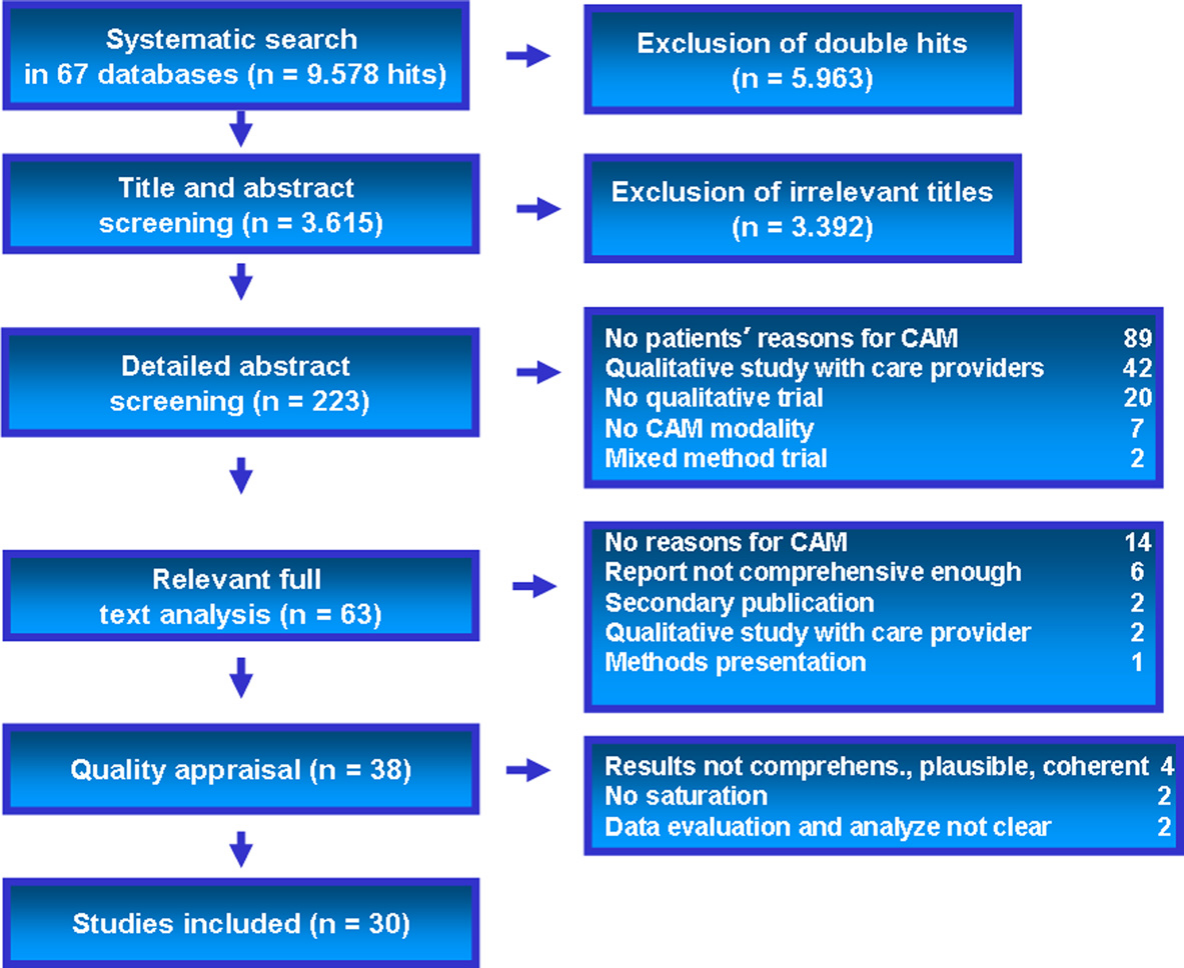
Abstract screening and full publication assessments.

A total of 9578 articles were found, duplicates were removed, and 3615 were screened on the basis of abstracts and titles. Sixty-three full publications were assessed for inclusion and exclusion criteria. Twenty-five publications were excluded after full text analysis and eight publications were excluded after the quality appraisal for reasons shown in Figure 1. The included studies were carried out almost exclusively in the United States, the United Kingdom and Australia and most literature included studies of cancer patients (Table 5). Because of the high number of included studies, a broad spectrum of patients from different countries using different CAM modalities could be obtained, and the research question could be answered more generally and not by dealing with special cases only.

**Table 5:** Publications included in the study

**Illness categorization**		**CAM treatment**		**Study country**		Study participants^*^	
General medicine	9	Mixed CAM	27	USA	11	Adults	15
Chronic disease	4	Mind-body (mediation, prayer)	2	UK	4	Females	5
Cancer	14	Body-based therapies (e.g.massage)	1	Australia, New Zealand	6	Males	5
HIV	3			Denmark, Norway	3	Older Females	1
				Canada	5	Adolescents	2
				China	1	Families including children	2

^*^All education levels including low level education.

### Results of the quality appraisal

The checklist of Behrens and Langer turned out to be a suitable tool of quality appraisal for the high volume of studies. The checklist was tested by the whole qualitative research group. With the detailed questions for the reviewers, it was possible to answer the standardized questions of Table 3 consecutively and with structure as well as compare the results between reviewers. The standardized questionnaire helped the reviewers to go back and reflect on their personal notes continuously. Based on the standard approach promoted by the Cochrane Collaboration and by The Joanna Briggs Institute (JBI-QARI), two reviewers (MS, BF) independently appraised the data critically [29] and reflected on any differences. The level of inter-rater agreement reached nearly 100% except for one case. In this case, one rater found that the questions were not open and that the data collection was oriented to the interests of that particular studied institution. This case was discussed and consensus reached with the third co-reviewer. As a show case, one excluded study was discussed in a joint colloquium meeting with the whole research group and the exclusion was confirmed. The monthly research colloquium consisted of 5–10 researchers of our institution in the field of CAM with backgrounds in medicine, psychology, philosophy and nutrition science research as well as qualitative research. Most researchers are performing primary qualitative research projects in CAM and projects for the development of decision aids. Figure 1 lists the final reasons for the exclusion of the appraised studies.

Those final reasons, however, represent a composite of various reasons for the exclusion. The appraisal scheme permitted assessment of methodological rigour and quality in the qualitative studies using individual quality indicators from Behrens and Langer. In the beginning of the meta-ethnographic process in particular, it implied the certainty that the results mentioned in the primary studies were plausible enough to develop a higher interpretation. For most of the criteria, the quality of excluded studies was generally low and consequently this led to their exclusion. When a study was excluded, the principal reason for the exclusion was noted. Because many studies were included in the synthesis, the resulting second-order constructs of the meta-ethnography seemed to be saturated. The themes from the excluded papers would not have altered the meta-synthesis. Theoretical study saturation was achieved because no new relevant data emerged regarding a category, either to extend or contradict it [48]. Included studies were conveyed to a spreadsheet for the meta-ethnographic procedure and we extracted the first-order themes from each paper. We then translated our key themes across all articles to determine second-order key themes. The findings from the translation and the resulting second-order themes and subthemes provided the foundation for synthesis of the included studies and the interpretive analysis. The results of the meta-ethnography with “concepts of individualized medicine from the perspective of patients using CAM” will be presented in a further publication.

## Discussion

This article demonstrates a case example of how qualitative research in the field of CAM studies was identified and critically appraised for conducting a meta-ethnography. To make qualitative results more accessible to clinicians and health policy decision makers, findings from related studies can be synthesized. The meta-synthesis methods vary widely, including the search for literature, the appraisal of methods and findings, and finally the synthesis of the existing knowledge [24]. The search methods in systematic reviews investigating solely RCTs are more or less established, but for qualitative research, an on-going evolution can be observed [14]. The Cochrane Qualitative Methods Group in its Study Collaboration Chapter 7 “Searching for Studies” exclusively described search techniques of qualitative research with the aim of developing methods for the inclusion of findings from qualitative research into systematic reviews [40]. The chapter seeks to build on the guidance of earlier chapters and on generic publications about “Searching for Studies” [49]. The search results shown in Table 4 are confirmed by findings from other qualitative systematic reviews [22,50,51]. The challenges of retrieving qualitative research are well-documented [50-52]. These include non-meaningful titles, a superficial depth of indexing, and poor description of the qualitative methods [51-53]. Poor indexing in databases like GREENPILOT or MedPilot and the diversity of qualitative research also made the development of search strategies for identifying qualitative studies difficult in our project [14]. The difference from other reported search strategies in the literature was our research question. Our research question asked for concepts of individualized medicine from the perspective of patients using CAM in general and not for a specific CAM treatment. We searched for “qualitative research” AND “CAM”, which provided abundant references. Plinkington (2007) found also that there is a “fine balance between achieving a search in the CAM field that is sufficiently sensitive to identify all the relevant studies and one that is sufficiently specific to avoid irrelevant information” [19]. For our meta-ethnographic study, it was important to search a range of databases. Four of the fourteen databases listed in Table 4 showed another single unique study not listed in PubMed. However, in contrast to Pilkington (2007), the specific CAM databases delivered no additional hits for the combination search term “qualitative research” AND “CAM” [19]. Combining the literature search for “qualitative studies” and “CAM” meant “carefully calibrating and recalibrating search strategies to ensure that data collection efforts yield more than narrowly redundant data” [21]. We found that the single broad search terms “qualitative research” or “qualitative studies” or “interviews” had the highest recall and precision. We wanted to ensure that very few qualitative articles were missed and used a sensitive search strategy, but for MedPilot and GREENPILOT the precision for this strategy was very low. Many hits needed to be sorted through to find articles on target and we suggest not using MedPilot and GREENPILOT for a search question combining “qualitative studies” AND “CAM”.

In most databases, the search term “patients’ reasons” could not be connected with the search string “qualitative research” and “complementary and alternative medicine”. Thus, the literature search question “patients’ reasons for CAM” was kept very broad with “complementary and alternative research” AND “qualitative research” and the analysis of hits turned out to be time consuming and complex [14]. As reported by Fleming et al. (2007), longer search strings and detailed key words were less specific and the broader text terms (qualitative research) or (qualitative studies) were more efficient in the search for specific literature [22]. Other published meta-ethnographies in CAM have also reported that the literature search was comprehensive, requiring analysis of numerous hits before starting the quality appraisal [35-38].

Murphy et al. (2003) searched biomedical databases on complementary medicine and came to a conclusion similar to ours, that searching databases on CAM-related topics is challenging because of the diversity in the use of vocabulary and of indexing procedures in different databases [54]. They suggested collaboration among indexers, authors, investigators and information specialists to develop standard terminology in CAM. Because the CAM literature is continuously growing, the dissemination of a controlled vocabulary will become even more important [54]. CAM trials should be uniformly indexed with a specific medical subject heading (MeSH) term such as “complementary medicine” in all databases as suggested by Bardia et al. (2006) [20].

The CAM databases did not deliver any support. CAM-QUEST® is a database for RCTs only; qualitative studies are not listed there. The CAM-QUEST**®-**database is designed as a search tool for people interested in reliable information on CAM. The aim of the databases is not to rate clinical studies but rather to point out that a large quantity of clinical research in the field of CAM exists. In addition, there is the possibility of performing a search in a large number of homeopathic case reports [45]. It is debatable whether a database like CAM-QUEST® should include qualitative studies, having in mind the value of such studies. Although PubMed also has its limitations (e. g., “CAM” often has a different meaning than “complementary and alternative medicine”), we received the most relevant hits in a short time frame. PubMed was shown to be the most effective database because hits were precise and abstracts could easily be obtained and screened. In addition, CINAHL and DIMDI produced precise hits and would have been reasonable databases for starting the search strategy. To reach a critical mass of literature in a search for “CAM” combined with “qualitative research”, the most effective strategy appears to be to use PubMed as the first database followed by CINHAL (or vice versa), and then continue with PsychINFO and DIMDI (which is another comprehensive database including many other databases). With these main databases, a substantial number can be reached without losing too much time with smaller and less efficient databases. It could then be tested whether saturation of findings has been achieved by analysing special CAM databases and/or by hand searches.

The assessment of the quality of qualitative research remains controversial. It is still a topic of discussion whether to appraise the methodology of qualitative studies at all, to employ one’s own qualitative criteria, or to apply quantitative methods. Keeping in mind the guidance of the Cochrane Group, at the beginning of the project we decided to make a considerable effort to search for suitable checklists. According to the Cochrane Qualitative Methods Group, reviewers need to decide for themselves which instrument appears to be most appropriate in the context of their review and use this judgement to determine their choice. Researchers with a quantitative background also need to consider an input from researchers who are familiar with qualitative research, even when an appraisal instrument is adapted for novices in the field [41].

We decided to use the checklist of Behrens and Langer from the University of Halle since qualitative research with its evidence-based emphasis on the field of nursing seemed to be the most suitable for the research question of patient expectations. However, it could be criticized that this German checklist was not comprehensive enough since we added three additional parameters and it was therefore not validated. The checklist has not been used so far in international published studies and is not mentioned in the Cochrane Qualitative Methods Group handbook. For those reasons, we compared the results of our appraisal with an international validated checklist from Tong et al. [31]. To increase reliability, we appraised the studies again with the supplemental questions from Tong et al. and came to the conclusion that all appraisal results were the same. Hence, we concluded that the checklist from Behrens and Langer is a suitable tool for qualitative research questions like patient perceptions or expectations. However, the checklist of Cohen & Crabtree or Tong et al. could also have been applied for our research question. Generally, we experienced that appraising each study was an important factor for our project when synthesising the studies afterwards. In particular, the plausibility of the data analyses and the reporting of themes seemed to be very important for performing the meta-ethnography afterwards.

Furthermore, the overall questions added from validated checklists to our adjusted checklist from Behrens and Langer (openness, inter-subjective validation and reflexivity) were only rarely reflected in the appraised publications (Table 3) and should be considered for reporting in future qualitative research publications.

## Conclusions

The aim of this publication was to present a case study of how to locate and appraise qualitative studies for the conduct of a meta-ethnography in the field of CAM. The example showed that the literature search and appraisal was a very comprehensive task with a large number of possible methods starting with the literature search and ending with the appraisal of existing papers. The findings of our comprehensive database research showed that CAM qualitative studies cannot be easily retrieved from CAM databases. CAM databases should increasingly include qualitative studies, given the value of such studies. PubMed, CINAHL and DIMDI delivered the most precise hits. Databases like GREEPILOT and MEDPILOT seemed less well-suited to searching for qualitative studies. Generally, appraisal of the included studies was a valid exercise, especially for plausibility, which is important for meta-ethnography. Because of the high number of included studies, a broad spectrum of patients in different countries using different CAM modalities could be obtained without the limitation of dealing with special cases only. Further research needs to validate existing methods, and guidance for certain research questions needs to be developed.

## Abbreviations

CAM: Complementary and Alternative Medicine; HTA: Health Technology Assessment; RCT: Randomized Controlled Trial

## Competing interests

The authors declare that they have no competing interests.

## Authors’ contributions

All authors contributed to the development of the manuscript. All authors read and approved the final manuscript.

## Pre-publication history

The pre-publication history for this paper can be accessed here:

http://www.biomedcentral.com/1472-6882/13/125/prepub
